# A roadmap to define and select aquatic biological traits at different scales of analysis

**DOI:** 10.1038/s41598-023-50146-9

**Published:** 2023-12-22

**Authors:** Teófilo Morim, Sofia Henriques, Rita Vasconcelos, Marina Dolbeth

**Affiliations:** 1 CIIMAR - Interdisciplinary Centre of Marine and Environmental Research, Novo Edifício do Terminal de Cruzeiros do Porto de Leixões, Avenida General Norton de Matos s/n, 4450-208 Matosinhos, Portugal; 2 IPMA – Instituto Português do Mar e da Atmosfera, Av. Dr. Alfredo Magalhães Ramalho 6, 1495-165 Algés, Portugal; 3 MARE - Marine and Environmental Sciences Centre & ARNET – Aquatic Research Infrastructure Network Associated Laboratory, Faculdade de Ciências da Universidade de Lisboa, Campo Grande, 1749-016 Lisbon, Portugal

**Keywords:** Ecology, Ecology, Biodiversity, Community ecology

## Abstract

Trait-based approaches are a powerful tool, as they not only improve understanding of ecological complexity and functioning but also allow comparison across different ecosystems and biogeographical regions. They may be used to unveil ecosystem processes and assess community structures, but their great potential becomes limited when dealing with scattered trait data and historically unstandardised trait nomenclature. The lack of standardisation allows authors to use the terminology of their preference, which inevitably leads to ambiguous misunderstandings and limits comparison between different studies. There have been some attempts to organise the trait vocabulary, but even these are mostly created from the perspective of a single ecosystem, which limits their applicability. In this work, we conducted a systematic literature review that identified and compiled 1127 traits across 37 datasets of fishes, invertebrates and zooplankton from freshwater, marine and transitional ecosystems. This dataset was then used to build on the Marine Species Traits Wiki and to propose a new, unified approach to a trait vocabulary based directly on readily available trait data. We propose a single standardised designation for all the different traits identified and provide a list of all the different synonyms commonly used for these traits. A roadmap to help the trait selection process is also provided, offering a guide through four main steps and important questions for choosing an adequate set of traits at the beginning of any study, which constitutes one of the main challenges in functional ecology research. Overall, this proposal will provide a solid baseline for tackling gaps in trait nomenclature and ensuring a clearer future for functional ecology studies.

## Introduction

Aquatic ecosystems are highly contrasting, from marine to freshwater, from deep water to intertidal and temporary freshwater habitats, from coastal to high altitudes, and from tropical to polar, in all-encompassing distinct environments and communities^1^. These ecosystems are threatened by increasing levels of disturbance caused by several drivers of change, such as overexploitation, water pollution, habitat loss and degradation, flow modifications and the introduction of invasive species^1–3^. Such threats have resulted in the change and loss of aquatic biodiversity at alarming rates^4,5^, affecting ecosystem functioning and the ecosystem’s capacity to supply ecosystem services^2,6,7^. Shedding light on the processes and mechanisms shaping community dynamics and how they respond to disturbances is of great importance since it may facilitate the creation of more effective management and conservation strategies^1,8^. To this end, trait-based and functional diversity approaches have been increasing in recent decades, as they provide information on the effects of species and communities on ecosystem functions and thus on how systems can function and respond to ecological change^9^. This can represent answers to pivotal questions in ecology, such as why species coexist, what affects this coexistence, what are the different ecological niches and, thus, their distribution.

Community ecology has traditionally relied on species-based approaches^10^. However, trait-based approaches have been used for over a century and can be more informative when used to complement more traditional approaches^11–13^ or even as an alternative, for instance, when studying the impact of low-to-moderate intensity drivers in populations and communities^9^. Examples of the added value of using traits include comparing patterns between geographical areas and/or different biogeographical regions that do not share common species to assess the ecological processes driving community composition. Traits can be defined as any morphological, physiological, or phenological feature measurable at the individual level, while functional traits are any trait which influences organismal performance^14,15^. Traits can be further divided into effect traits, defined as how any given species affects ecosystem properties, and response traits, defined as how it responds to a disturbance or environmental change^16^. In addition, traits might be indicators of ecosystem functioning and may facilitate the identification of the mechanisms and drivers generating community patterns and changes^8,14,17,18^. Furthermore, conclusions may be transferable among ecosystems across biogeographic regions, being independent of species identities, thus, allowing for more generalised knowledge of community patterns and changes^14,19,20^.

There is a large body of work on species’ traits, with several publications and databases providing trait data compilations (e.g., BIOTIC^21^; freshwaterecology.info^22^; FishBase^23^) that aim to organise the large amount of information produced and published during the last century. The increase in data availability^24^ and computational tools have promoted the rise of trait-based approaches^10^, which further evidences the need for collaborative work on trait data compilations for a variety of biological groups^25,26^. Despite considerable efforts (e.g.^24^), increased transparency and data access, trait data remain scattered and their nomenclature historically unstandardised^11,12,27^, which hampers their use to carry out traits’ metanalyses studies, comparative studies or understanding the relevance of such approaches across different studies. Different authors may utilise misleading terms and definitions that may be associated with distinct concepts, a situation further aggravated by publications written in different languages. In this context, having access to additional information, such as both trait and modalities’ definitions, along with a proposed synonym, may be crucial to avoid misunderstandings, especially for researchers who are less familiar with working with traits.

The lack of standardisation, clearly present within trait-based studies, can be overcome by employing different approaches. One can discuss the semantics of what constitutes and defines a trait^13,28^ and their mathematical use^28^, and one can organise and propose a standard template for future databases to follow^11^, or one can tackle the multitude of terms and definitions used to define traits and functional traits by developing thesaurus, glossaries or vocabularies (e.g.^21,29–31^). Notably, ^29^ provided a baseline for the creation of a standard vocabulary for marine species traits, which is currently implemented in the WoRMS database^32^ and available under a Wikipedia format^33^. Additionally, it is an important tool for the scientific community to build on towards attaining a unified terminology and reducing nomenclature misuse. However, restricting this comprehensive vocabulary to “marine species” might reflect that many aquatic community ecology studies address their questions from a single-type ecosystem perspective. We believe the next step towards a unified trait terminology is making it applicable across different ecosystems. For instance, ^34^ acknowledged the importance of cross-compatibility between ecosystems by establishing a correspondence between their marine deep-sea traits database and other databases, highlighting that apparently similar traits may be associated with different terminologies.

Other obstacles regarding trait-based approaches span from as early as the planning of the research itself^9^ since selecting and defining traits to include in a community ecology study can also be very subjective. Despite directly impacting the results, trait selection remains unstandardised and, along with how traits are measured, depends completely on the researcher’s approach. Species displaying unclear or variable behaviour represent an equally subjective aspect, where expert judgement is frequently sought after to help categorise traits for poorly described species and may result in different categorisations according to who performed them.

In this context, we build on the previously published trait vocabularies by^29^, compiling trait databases available from the literature for three taxonomic groups—fish, aquatic invertebrates and zooplankton, and across aquatic ecosystems (i.e., freshwater, estuarine and marine). Identifying the traits currently described in scientific publications and online datasets allowed us to determine the terms and designations used to classify them, and to provide information on the availability of traits information based on the number of classified taxa (important to further select the traits to be used) as well as to detect inconsistencies regarding terminology. These data were then utilised to propose a new holistic trait hierarchy/vocabulary compatible across different aquatic ecosystems, ensuring a clearer and more coherent use for future studies in aquatic functional ecology. Thus, we aim to harmonise the concepts used to describe traits, in general, updating an existing and used nomenclature, and providing guidance on trait selection based on their informativeness versus data availability in the literature. For this, we also included a brief section on trait selection along with a roadmap exemplifying the main steps necessary to go through the abovementioned processes.

## Materials and methods

### Literature search

A systematic literature search was performed in May 2020 to identify public databases compiling information on traits of fish, aquatic invertebrates and zooplankton. We utilised Web of Science, running the following Boolean combination in the “topic” field tag: (fish* OR macroinvertebrate* OR zooplankton OR fauna* OR marine OR freshwater OR brackish water OR saltwater) AND (ecological OR biological OR functional) AND trait* AND (database* OR dataset*). The search was conducted in all Web of Science databases and for an all-years timespan up to May 2020.

The results were then manually screened for eligibility. Peer-reviewed studies meeting the following criteria were selected for inclusion in the present work: (i) taxa must belong to our target taxonomic groups (fish, aquatic invertebrates, and zooplankton), (ii) trait data must be readily available (data file available via a database dedicated website, in-text table, supplementary material or accessible via R package) and (iii) more than 50 taxa must be covered, to limit the number of entries that might be too context-dependent and avoid data replication. If the study failed one of these criteria, we excluded the study.

Since we aim to report on the traits that are available and easily accessible in the literature, we have deliberately excluded publications when data were only available on request or where the authors did not indicate, for example, the meaning of the abbreviations for taxa or traits. We also excluded studies focused exclusively on paleobiology or toxicology-related traits. Furthermore, in many cases, multiple studies use the same previously published database for their analyses, such as^22,23,35^, resulting in the pseudoreplication of information. To avoid this, we eliminated these subsets, maintaining only the original complete database. Thereby, we removed studies which made use of (i) an integral copy of other databases, (ii) other databases as the main source, further completing its gaps with information from the literature, or (iii) subsets of other databases. We understand the information in these subsets might appear rearranged, better organised or easier to process, but we removed them as we aim to compile the original sources of traits information (e.g.^36,37^). Finally, if a given database was published and subsequently updated, we kept only the most recent version.

### Trait categorisation, definition and standardisation

Each study's taxa x trait tables were retrieved and all the traits described in each database were characterised by collecting several descriptors, such as name and definition according to the author, geographical region and environment of the study, name in the original dataset file, the number of taxa described, measurement type, trait modalities or measurement unit, data accessibility, among others (complete list and examples in Table 1). Many studies only provide an estimate of the number of taxa for the entire traits dataset, without providing the number of taxa for each trait individually (i.e., there might be a mismatch between trait data completeness per species and the number of taxa described in the complete dataset). Thus, we calculated the number of different taxa with information at any taxonomical level for every trait from the datasets we compiled to allow the comparison of traits’ availability. Most of the entries described were for species-level (more than half), followed by genus in a lower proportion or for specific datasets (e.g.^38^), and occasional entries for family or order. Our estimates varied a bit when compared with the counts provided by some of the authors, which is most likely due to assessing slightly different dataset versions in case it was updated recently, or by employing different counting criteria regarding the multiple taxonomical levels described in some of the studies. Nevertheless, we do not consider these differences relevant, as our taxa counts are only meant to indicate a broad idea of the distribution and availability of trait information in the literature. The measurement type refers to how the trait is quantified and can take a numerical (for continuous numerical traits), or a categorical value. We also add the possibility of text for when the trait is further discriminated as text (e.g., all food items from a diet). The categorical traits include (1) categorical, for multi-categorical traits (> 2 modalities), ordered (e.g., small, medium, large) or unordered (e.g., detritivore, herbivore, omnivore); (2) binary, when restricted to 2 modalities (e.g., yes/no or true/false traits), and (3) fuzzy coding when species can be assigned to more than one modality with an affinity score.

**Table 1:** Information collected in each dataset compiled from the systematic review of literature trait data sources, with a description of the data collected, their description and an example of the data collected for each of the variable (see *Trait_Sources&Measurements* dataset in Supplementary Information).

Information collected (variables)	Description	Example of measurements for each variable
Environment	Main environment classification as freshwater, marine or estuarine	Freshwater
Main taxa group	Main taxa classification as fish, invertebrates and/or zooplankton	Fish
Region	If worldwide or in a specific world region	Arctic
Trait descriptor	Trait descriptor classification according to^33^, as Biological, Distribution or Ecological	Biological
Traits classification	Body form, Body size, Body pigmentation…	Body size
Trait standardised name	Trait standardised name, after reviewing all data sources and attributing a common designation for the synonyms, also considering their definition (Table 2)	Body length
Trait name (original author)	The trait name provided by the author (unstandardised name)	Body size (maximum body length)
Trait name in data file	Trait name utilised in the original dataset file	Length
Number of taxa	Estimate of the number of taxa described for each trait	52
Definition (original author)	Definition provided by the author, when available	Maximum body length
Measurement type	Type of measurement, if numerical, text or as categorical (simple, binary, or as fuzzy coding)	Numerical
Measurement/modality	Available measurements, including quantitative units (i.e., mm, cm…) or qualitative (i.e., modalities of the category, such as small, medium, large). For the modalities, it includes their definition when available	cm
Author	The authors of the database	Frainer et al. (2017)
Database name	If available as a database and which one (e.g., BIOTIC, FishBase…); if not, described as NA: non-available	NA
Accessibility	Where the trait data is accessible and the file’s format (e.g., database dedicated website—csv, supplementary material—docx…)	Table in supplementary material
Source	Link for the publication or database source if available	https://www.univie.ac.at/arctictraits/

Details of the selected datasets are available in section "Literature search".

Traits were then sorted along different hierarchical levels from the Marine Species Traits Vocabulary (www.marinespecies.org/traits/wiki^33^) that follows the nomenclature initially proposed by^29^. We opted to use this Wiki’s structure as the baseline for constructing our new nomenclature, not only due to its well thought structure but also because it is already a vocabulary familiar within the scientific community, as it is currently implemented within the WoRMS platform^32^. Additionally, the fact that WoRMS information is continually being refined and updated may also allow for a simpler and faster implementation of new trait nomenclature standardisation proposals published in the future. Adjustments were made to the original Wiki structure available online (1) considering some sections are incomplete (e.g., Physiology which had no information assigned) and (2) to accommodate all newly collected traits. Establishing a correspondence between traits and Wiki implied adding entries to account for all new traits which had not been previously included and removing previously existing traits if there was not any data available for them. Some of the original traits proposed in^29^ classification may serve as proxies or reflect a combination of the traits that would actually allow species to exist, tolerate or even thrive under certain environmental conditions (e.g. biogeographic distribution, salinity preference, among others). For example, biogeographic distribution reflects the interplay between physiology, movement/dispersal ability and environmental conditions, whereas salinity preference lies between physiology and environmental conditions. We have also reviewed these proxies of traits in this work, as part of the original trait nomenclature^29^ and because authors still use them as reflecting the species performance in the environment^39,40^, as their information is easier to collect^9^. Additionally, our trait datasets focused on fauna, thereby we did not consider any traits specific to flora (plants, seaweeds or microalgae). Nevertheless, there is some work on standardising plant traits and protocols for their measurements^15,41,42^ and datasets (e.g. TRYdatabase: www.try-db.org), while for seaweeds recent work with datasets (www.algaetraits.org^43^) has compiled information on their traits and how to measure them (e.g., seaweedtraits.github.io). While some traits are specific to flora (e.g. photosynthesis, light uptake), others are common, such as size, growth rate, respiration and others.

The present work focuses on three trait descriptors proposed by^29^: “Biological descriptors”, describing a species' body size, its life history characteristics or physiology aspects; “Distribution descriptors”, describing the “environment and habitat in which a species lives, and its spatial distribution by geography, depth, and time”; and “Ecological descriptors”, describing the species habit (including ﻿external appearance or form and behaviour) and feeding. In this classification, the “Species Importance to Society” main group was excluded. All traits were sorted along the hierarchical levels of the vocabulary, starting by placing them in one of these three trait descriptors, followed by the Traits classification and Trait standardised name. A key step for sorting all traits accordingly was to identify and group similar traits that are likely to translate the same information but may be referred to with distinct names. Given the overall lack of guidelines for employing cohesive trait terminology, different authors commonly refer to the same trait with several names (e.g. life span, life duration, longevity). Naturally, this also means there will be cases in which authors refer to a trait using a name more typically associated with other trait modalities or trait information, resulting in some trait designations becoming misleading. To overcome this, we first assessed each trait’s definition, measurements/set of modalities and their definitions when available, guaranteeing we understand clearly which information is truly being conveyed. The synonyms (here referring to all different terms that translate the same trait information) were retrieved from the compiled databases and correspond to the trait name used by the author in their *taxa x trait* tables. Once grouped, synonyms were then associated with one Trait standardised name. We further labelled synonyms as “Misleading” if they were ambiguous (e.g., “feeding ecology” for being too general, “feeding type or habit”, which suggests a feeding method rather than a food type), and as “Specificities” in case they represent a very specific aspect from the corresponding Trait standardised name. The complete list of traits and classifications, their definitions, synonyms, measurement options based on the datasets and literature references for the definitions are available in the *Standardized_traits* dataset (Supplementary material). This list was based on the information available in the literature, from the selected databases that matched our criteria, all compiled in the *Trait_Sources&Measurements* dataset (Supplementary Information).

### Alluvial diagrams and flowchart

To aid the visualisation of the compiled datasets, we plotted three alluvial diagrams, each corresponding to one of the three trait descriptors: Biological descriptors, Distribution descriptors, or Ecological descriptors. The maximum number of taxa for each standardised name trait was sorted along three levels of information: Descriptors, Traits classification, and Trait standardised name (Table 1). A dendrogram was additionally used to plot our entire dataset, displaying the distribution of the number of traits along the hierarchy and allowing for easier navigation of our hierarchy’s different levels. Finally, we constructed a flowchart describing the main steps involved in the process of choosing which traits should be analysed in each study, simultaneously working as a roadmap for any user interested in using this study’s data compilation (*Trait_Sources&Measurements* dataset). The alluvial and dendrogram plots were created with the data visualisation tool Raw Graphs (www.rawgraphs.io) and the flowchart with Draw.io (www.drawio.com). Figures were then joined with Adobe Illustrator.

## Standardisation of the trait nomenclature

As previously mentioned, despite several attempts to reach a general consensus for traits’ nomenclature (e.g.^21,29,33^), current traits designations remain full of misunderstandings with an overall lack of organisation^27^. By reviewing the terms utilised in multiple databases concerning different aquatic ecosystems, we were able to uncover traits with conflicting nomenclature. For instance, a trait commonly poorly defined is the Food type/Diet (e.g., carnivore, herbivore, omnivore), which can be found in the literature referred to as “diet”^44^, “trophic mode”^34^, “feeding habit”^45^ or “feeding diet”^22^. The lack of consistency across different studies proves itself as a powerful obstacle, making it extremely difficult for anyone to properly search and find traits for their analyses in a time–cost effective manner. In addition, a single nomenclature is the first step towards the standardised use of traits and to the development of a more comprehensive global trait database for aquatic ecosystems, both of which constitute fundamental steps to improve the comparison between different studies, spatial and temporal scales, as well as biological groups.

The present study tackles these issues by using two different approaches. First, we built on the hierarchical classification started by^29^ by adapting its current Wikipedia version^33^ to render it suitable not only for marine traits but also for freshwater and transition ecosystems. Secondly, to reduce the multitude of terms in the literature, we organised and grouped similar terms, proposing a single standardised name for each trait, according to its significance and modalities, and stating its different designations found in the literature (i.e., synonyms, as described above). New traits were added when there was no correspondence with the traits already described by^33^, and definitions were adapted to broaden their meaning. Despite all modifications, we aimed to make the lowest number of possible changes, which we understand will facilitate its implementation in future studies.

### Trait proposed nomenclature 

The revision and nomenclature adaptation of Biological, Distribution and Ecological descriptors proposed by^29^ resulted in 18 trait classifications for each, further divided into 59 trait-standardised names. Of the latter, 23 traits were newly added to fill in the information gaps from the previous Marine Species Traits Wiki (Table 2). These new traits are based on our review as they were not discriminated in the original dataset.

**Table 2:** Description of the different synonyms compiled from the literature, distributed on three hierarchical levels: (1) Descriptor, (2) Traits classification and (3) Trait standardised name.

Descriptor	Traits classification	Trait standardised name	Synonyms
Biological	Body size	Body lengthª	Body length; Body size; Known length; Length or length type; Measured length; Potential size; Reference length; Size or Size type; Total length Specificities: Body width; Fork length; Larval length; Length at birth; Measured height; Pre-flexion/ Post-flexion—Reference length; Prosome length; Size at birth; Size at first feeding; Standard length
		Body massª	Mass; Micro gram carbon; Weight
		Colony sizeª	Colony diameter or area; Colony mass or weight
	Life history	Age at maturity	Age at first reproduction; Average age of mothers in populations; Female age at maturity; Female maturity; Male maturity Misleading: Generation time; Age range
		Body size at maturityª	Length at maturity; Size at maturity; Female size at maturity Specificities: Fecundity-length relationship
		Diel activity patternª	Activity period
		Egg incubation timeª	Gestation/incubation; Hatch time; Incubation period
		Egg or propagule size	Egg axial length; Egg diameter; Egg diameter (outer, maximum); Egg Size (mean); Mature egg diameter; Oocyte size at maturity; Propagule size on release
		Egg type and Yolk characteristicsª	Larval yolk sac; Shape and pigmentation of yolk-sac Misleading: Spawning strategy
		Emergence patternsª	Emergence behaviour; Emergence duration; Emergence period; Emergence season; Emergence synchronization; Flight period
		Fecundity	Clutch size; Eggs per area; Fecundity: relative, absolute, scale; Litter/Clutch size; Maximum number of descendants per reproductive cycle; Number of descendants; Number of eggs or offspring or oocytes Specificities: Polyp fecundity; Mesentery fecundity
		Fertilisation	Fertilization type; Mode of fertilization; Reproduction habitat; Reproduction location; Spawning; Spawning habitat; Spawning strategy Misleading: Mode of larval development; Reproductive guild Specificities: Sperm type
		Fertilised Egg/Larval settlement locationª	Habitat type of settlement/early development; Oviposition site; Reproduction habitat; Substrate type of settlement Misleading: Egg/egg mass; Oviposition behavior; Parental care; Reproduction; Reproduction type; Reproductive guild Specificities: Eggs cemented
		Generation time	Interbirth interval
		Larval and juvenile development	Development type; Developmental mechanism; Larval and juvenile development; Larval development; Larval mode of development; Location of parental care; Parental care; Parental care/Brood protection Misleading: Larval area reproduction; Reproduction mode; Reproductive guild; Place of development
		Larval settlement period and stage durationª	Development duration; Larvae occurrence (time, when); Larval settlement period; Larval stage duration; Larval development cycle; Length of larval development; Presence of larvae (time, when); Time larvae spend in plankton Misleading: Development pattern
		Life history—miscellaneousª	Mortality rate: initial mortality rate, mortality rate doubling time
		Life span	Adult life duration; Adult life span; Life cycle duration; Life duration; Longevity; Maximum age; Maximum longevity
		Reproductive frequency	Batch Spawner; Number of Litters/Clutches per year; Maximum number of reproductive cycles; Number of reproductive cycles per individual; Potential number of cycles per year; Reproduction strategy; Reproductive life cycles per year; Reproductive type; Spawning frequency; Voltinism
		Reproductive season	Spawning season; Spawning period; Spawn time; Season length Oviposition period
		Reproductive type	Mode of reproduction; Reproductive system; Reproductive technique; Mating system Misleading: Reproduction Specificities: Asexual reproduction; Sexual system; Hermaphroditism; Gonochorism; Epitoky
	Life stages	Life stage	Aquatic stages; Number aquatic life stages
	Physiology	Elemental compositionª	Specificities: Carbon content, C corporal content, Body C; Nitrogen content, N corporal content; Phosphorus content, P corporal content, Body P; C:N ratio, Body CN, Body N; N:P ratio, N:P molar ratio in corporal content, Body NP; Lipid content, Lipid in corporal content; Storage organ of fats; Fats storage; Protein content, Protein biomass; Biomass of protein per unit of skeletal surface area;Lipid in the coral holobiont tissue per unit of skeletal surface area
		Excretion rate/ratioª	Individual excretion rate; Mass-specific excretion rate; Evacuation rate Specificities: Ammonia excretion rate; Excreted N:P; N or P excretion rate; N:P molar ratio in excretion; Phosphate excretion rate
		Life history rates ª	Specificities: Calcification rate; Development rate; RNA:DNA ratio (as proxy of growth); Growth; Growth rate; growth parameters (Loo, r, K, t0); Specific growth; Ration (% BWD)—Percent body weight per day
		Metabolic ratesª	Specificities: Clearance rate; Food consumption rate; Oxygen consumption; Respiration rate (mass-specific, dark); Specific respiration; Effect of activity on metabolic rate
		Metabolism—miscellaneousª	Myelination
		Respiration modeª	Respiratory method Specificities: Haemoglobin
Distribution	Depth	Depth range	Depth range; Depth preferences; Optimal depth
	Elevation	Elevation	Altitude; Altitude preference
	Environment	Environment	Ecosystem type Misleading: Habitat; Salinity preference/preferendum; Salinity tolerance
	Geography	Biogeographical distributionª	Basin region/Catchment region/Ocean Basin; Biogeographical zone; Bioregion; Climate region; Distribution (global, temperate, …); Ecoregional distribution; Geographical zone or region; Locality; Range size; Zoogeography Specificities: Ecoregion-endemism; Micro-endemism; Northern/Southern/Western-most range edge
	Habitat	Environmental position	Habitat/Lifestyle; Habitat affinity Misleading: Functional group; Water column position; Vertical habitat position
		Physiography	Distribution (e.g., Brackishwater, Freshwater); Ecosystem type; General/gross Habitat; General/gross Habitat; Habitat preference or association; Hydrological preference; Physiographic feature or type; Transversal distribution; Water zone; Waterbody type Specificities: Stream size association or preference; Transversal distribution along stream; Channel
		River longitudinal profileª	Hydrological preference; Lateral habitat position water column; Stream/River longitudinal distribution; Stream/River zonation preference Specificities: Potamal preference; Rhithral preference
		Salinity (regime)	Salinity; Salinity range; Salinity tolerance; Survival salinity
		Substratum	Habitat type; Microhabitat/substrate preference; Microhabitat/substrate preference; Seabed type; Substrate association, type, affinity or preferendum; Substratum affinity; Zonation (e.g., Hydrothermal vents) Specificities: Special habitats (e.g., beds, coral reefs…); Substrate—Soft Bottom or hard bottom; Exposure (to habitat)
		Tidal streams and water flow	Current optima range or rank; Current preference; Current velocity preference; Fluvial preference; Habitat Rheo; Rheophily or Rheo índex; Water flow rate
		Tolerance	Ambi Index or AMBI ecological group; Sensitive species; Tolerance (e.g., to organic enrichment, to pollutants, to silt); Tolerance level; Trophic status preference or preferendum; Turbidy/Water clarity tolerance
		Wave exposure	Degree of wave action (definitions); Wave exposure preference
	Vertical biological zone (or zonation)	Vertical biological zone (or zonation)	Depth zonation; Distribution—Marine (e.g., neritic, oceanic); Pelagic layer; Zonation
Ecological	Body form	Body shape	Body form; Body shape (lateral, case retreat); Cross section; Growth form (typical, Veron, Wallace); Growth outline type; Shape factor Specificities: Colony shape factor; Number of septa per corallite; Striking shape; Tissue thickness
		Body structures	Morphological adaptations; Skeleton; Striking feature; Supporting structures and enclosures; Vertebrae Specificities: Adipose fin; Armor, Spinal armature; Attributes of fins; Calcification; Dorsal head profile; Finlets; Fins number; Gas bladder; Lateral lines; Number of myomeres; Position fins; Scales (type, in or below lateral line, in lateral series, around caudal peduncle, …); Shape of gut; Soft-rays; Spines; Tube/burrow material, Tube construction; Type of eyes
		Flexibility	Body flexibility
		Fragility	NA
		Reproduction-related body formª	Different appearance during breeding; Different colours during breeding; Different morphology during breeding; Sexual metamorphosis; Specialized organs
	Body pigmentation	Colourª	Pigmentation; Melanophores Specificities: Rows
	Ecological Interaction	Habitat modification	Bioturbation; Ecosystem engineering; Foundation Species; Habitat Complexity; Sediment particle reworking Misleading: Habit Specificities: Habitat specialist
	Ecophysiology	Environmental parameters	Climate change affected species, climate change vulnerability score; Acclimation offset; Metabolic type; Oxygen: saturation preferences; tolerance; lethal; Hydroaerophily; pH: preference/preferendum; tolerance; acid class Temperature: thermal tolerance; survival temperature; optimal; preference; lethal; rank; indicator; reproduction; emergence; critical limit; extreme episodic; thermophily
		Resistance formª	Resilient strategies Specificities: Ability to survive desiccation; Ability to temporarily exit water; Diapause; Hibernation/Hibernation phase/instar; Resistance/resilience to droughts; Resting eggs
	Feeding	Feeding apparatus	Feeding structure Specificities: Barbels; Gill rakers; Position of mouth/jaws; Type of mouth
		Feeding Method/Behaviour	Feeding; Feeding behaviour; Feeding habitat; Feeding habits; Feeding location; Feeding mode (primary or secondary); Feeding strategy; Feeding type; Functional feeding groups Specificities: Hunting strategy
		Food Type/Diet	Chemosynthesis-obligate; Diet; diet composition; Dietary preferences; Energy source; Food name, type or size; Main food; Food; Dominant food; Nutritional Source; Trophic ecology; Trophic group; Trophic guild; Trophic level; Trophic mode Misleading: Feeding ecology; Feeding type; Feeding habit; Feeding method Specificities: Number links to predators/preys of different functional groups; Predator stage or predated by
	Mode of life	Dependency/Association	Associations; Dependency Misleading: Sociability Specificities: Parasitism; Symbiont (position, subclade, clade); Symbiodinium (density, clade, in propagules); Zooxanthellate
		Sociability	Coloniality; Gregariousness; Relation inter individuals; Sociability Misleading: Association; Occurrence in large quantities
	Morphometrics	Body parts sizeª	Specificities: Anal fin length and height; Aspect ratio of caudal fin; Axial length; Body depth at the level of the pectoral fin Insertion; Brain weight; Caudal fin length; Caudal peduncle minimal depth; Distance between centre of the eye to bottom of the head; Distance between insertion of the pectoral fin to Bottom of the body; Distance from top of the mouth to bottom of the head; Encephalization coefficient (brain weight / body weight); Eye diameter; Eye size; First dorsal fin length/ Second dorsal fin length and height; Forewing length; Head length, head depth; Lens diameter; Pectoral fin length; Pelvic fin length; Pre-flexion/Post-flexion—Depth at anus; Depth at pectorals; Preanal length; Pre-flexion/Post-flexion/Flexion—Preorbital length; Prepectoral length; Diameter of eye, Depth of eye; Pre-orbital length; Pre-pectoral; Pre-pelvic; Preanal Length (i.e., from snout to anus); Pre-dorsal length; Pupil diameter
	Movement	Dispersion	Dispersal capacity; Dispersal distance; Dispersal potential (incl. female dispersal, larvae and adult); Dissemination potential; Distance travelled in aerial habitat; Distance travelled in aquatic habitat; Drift
		Migration	Seasonal migration; Migratory; Type of migration; Diel vertical migration
		Mobility	Locomotion and substrate; Locomotion type; Mobility (Larvae or Adult); Movement (larvae or adult); Movement type; Relative Mobility; Speed Misleading: Substrate relation; Life type Specificities: Attachment; Crawling rate; Flying strength; Habit (primary or secondary); Living habit; Substrate attachment; Swimming (Mode, type); Swimming ability; Swimming speed

Synonyms may be further classified as “Misleading” if their name is typically associated with a different trait other than their matching Trait standardised name, and “Specificities” if they represent a very particular aspect of the corresponding Trait standardised name. All definitions and measurement examples for each Trait standardised name are available in an extended version of this table in the *Standardized_traits* dataset in the Supplementary Information, which also includes all the trait measurement options found in the literature, those mentioned in the^33^ original Wiki (when available), source references, the number of studies/data entries for each trait, and the minimum and maximum number of taxa described per study.

^a^Newly added traits which were absent from^33^ original Wiki.

More specifically, the four trait classifications defined by the^33^ for the Biological descriptors and 11 of the trait standardised names remained unaltered (Table 2). Then, four entries combined two previously distinct traits: (1) “Fecundity” combined “Fecundity and Clutch size”, as the latter also refers to the number of laid eggs (thus fecundity) for organisms that produce batches; (2) “Larval juvenile development” combined “Larval juvenile development”, the existence of “Parental care” and the “Brooding behaviour”, as parental care and brooding refer to particularities of the eggs/larval development; (3) “Larval settlement period and stage duration” merged “Larval settlement period” and “Duration of the larval stage” since both refer to time-based information over which larval settlement occurs; and (4) “Fertilised egg/larval settlement location” merged “Egg, egg mass or clutch location” and “Larval settlement substrate” as these are often mentioned simultaneously. In the Biological descriptors group, 17 new Standardised trait names were added, from which seven new traits were placed in “Life history” and six in “Physiology” (which had no information assigned in^33^). “Life history—miscellaneous” is one of the new additions and compiles trait information on life history which did not fall into the previously defined standardised names, such as “Spawning aggregation” or “Metabolic type”, respectively (Table 2). “Dormancy/diapause stage” and “Larval or juvenile dispersal potential”, two traits previously assigned as Biological—Life history on^33^ were moved to the Ecological group since they represent specificities of Ecophysiology and Movement (namely “Resistance form” and “Dispersion”, Table 2). “Gamete type” and “Life cycle” were removed as we did not find any information available on these from the retrieved datasets. All definitions found in the Biological group were either newly added or adapted from^33^ to better represent the information provided by traits, except “Generation time” and “Life stage” which remained unchanged.

We reduced the Distribution’s trait classifications to six, removing “Habitat preferences” and “Province”, while adding “Geography”. The trait “Province” was then merged with “Vertical Biological zone (or zonation)” as their information is often combined in the literature. “Biogeographical distribution” and “River longitudinal profile” were the only standardised trait names further added to the Distribution group, and eight definitions were either altered or newly defined.

The third and final trait group, the Ecological descriptors, saw the addition of “Body pigmentation” and “Morphometrics” to the six trait classifications previously listed in the^33^. These two new classifications bared each one a new standardised name, respectively, “Colour and Body parts size”. Regarding the “Body form” classification, we renamed Supporting structures and enclosures to Body structures, allowing for a broader definition, and further included Reproduction-related body form. We further propose a new organisation for the Ecophysiology classification. First, we included here “Environmental parameters”, which were created to combine *traits* such as “Climate change affected species”, “Metabolic type”, “pH”, “Oxygen and Temperature optimal, preferable, tolerable, or lethal values” (*Standardized_traits* and *Trait_Sources&Measurements* datasets). The last two were previously described as “De-oxygenation tolerance” and “Temperature range tolerated”. Secondly, we moved “Growth rate” from Ecophysiology to Biological—Physiology, which was renamed as “Life history rates”, as it reflects an intrinsic physiologic characteristic of the individual, despite the possibility of being affected by the local environment or the conditions to which it was measured (if experimental). Finally, we added the “Resistance form” trait (previously named “Dormancy/diapause stage” and placed it in the Biological group-Life history classification) as it allows a broader definition and inclusion of other resistance traits. “Dependency”, classified under “Mode of life”, was renamed as “Dependency/Association”. Within the Movement classification, the new “Dispersion” trait combines the former Biological trait “Larval or juvenile dispersal potential” and the Ecological trait “Dispersal potential (adult)”, and “Migration (Migratory)” was renamed as “Migration”. Furthermore, three traits were removed, “Growth form (or type)” and “Height (above substratum)”—both previously part of “Body form”—and “Support” from “Mode of life”. Lastly, 13 trait definitions included in this group were either added new or adapted from the original^33^ definitions.

As previously mentioned, using different terms when referring to a single trait is common. Grouping the synonyms collected during the literature review exposes this practice very clearly and highlights the importance of addressing it with new nomenclature proposals (Table 2). Most trait standardised names are associated with more than one term, it being a regular synonym, a misleading term, or a trait specificity, with some traits having up to 15 different synonyms (e.g., “Reproductive frequency”, “Food type/Diet”). Additionally, we identified 14 different trait standardised names affected by misleading designations (Table 2, *Standardized_traits* dataset). From these, half were classified under Life history, associated with Reproduction-associated traits, such as “Age at maturity”, “Fertilisation” or “Larval and juvenile development”, the rest being Habitat-related traits, Feeding-related traits, and traits from “Mode of life” and “Movement”.

### Availability of trait data for the proposed standardisation

**Figure 1:**
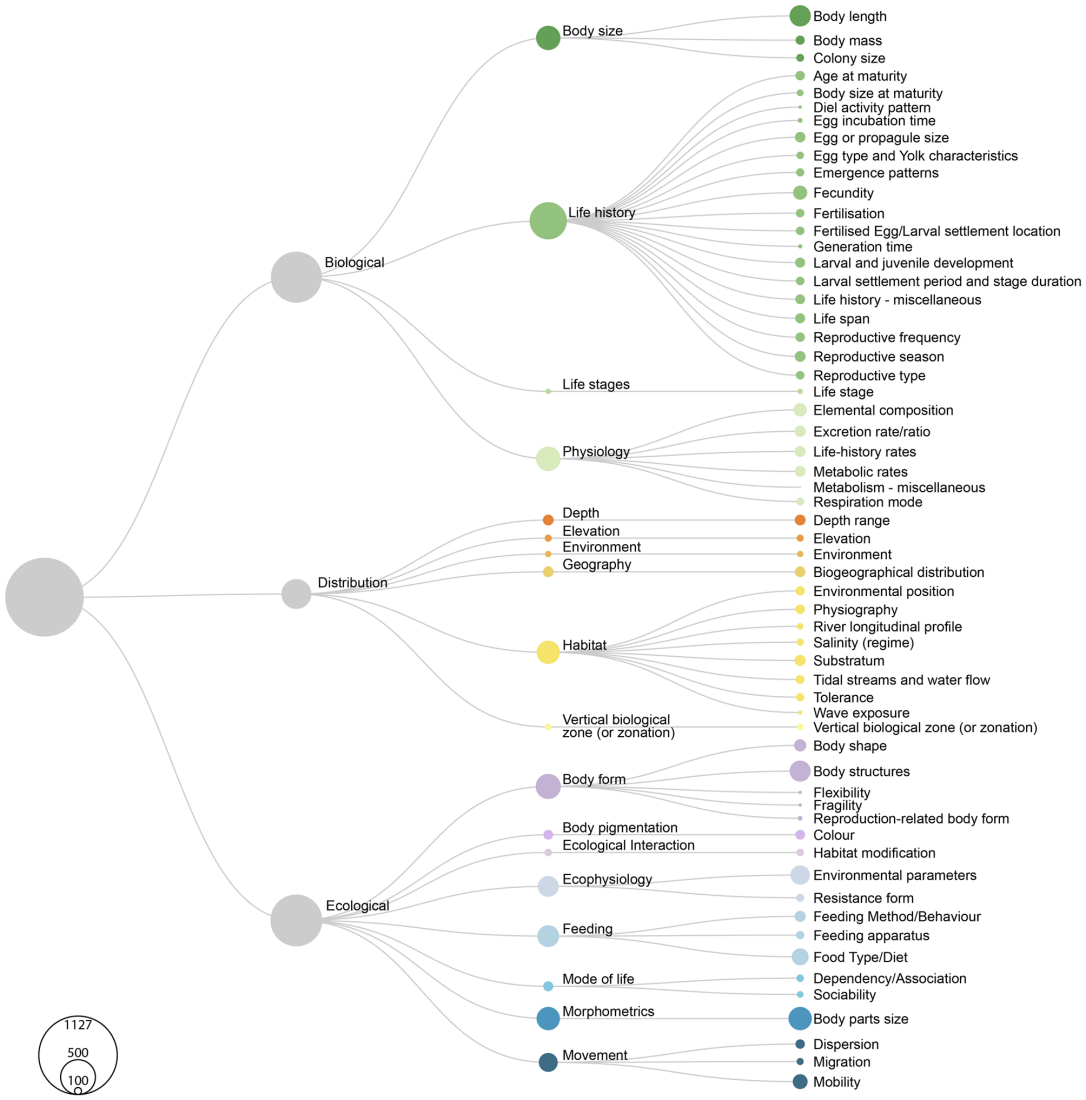
Distribution of the number of traits for aquatic fauna (fish, invertebrates and zooplankton), at the three hierarchical levels: Trait descriptors, Traits classification and Trait standardised name. The size of the circles at each node is proportional to the number of studies for which databases with information on the trait are available.

Our systematic literature review yielded 607 results, from which only 37 *taxa x trait* datasets matched our search criteria (Fig. 1). These can be separated into two groups, one for trait data focused on a single taxonomic group and a second for datasets with two or more taxonomic groups. The former gathered mostly data on fish (11 datasets) and invertebrates (14 datasets), with only four focused on zooplankton (we are here considering^22^ as two different sets as they have their data separated by taxa groups). The remaining multi-taxa databases divided into “Fish and Invertebrates” (3 datasets) and “Fish, Invertebrates and Zooplankton” (6 datasets).

As aforementioned, aquatic ecology tends to separate studies on freshwater and marine ecosystems, a trend which is reflected in the proportion of freshwater, marine and multi-environment databases. Only a third of the results provide information for more than a single environment, this being a combination of freshwater, marine and/or transitional ecosystems (30%). Exclusively freshwater or marine environments follow a similar proportion, accounting for respectively 32% and 38% each.

Due to the considerable variability associated with the region assessed in the databases, both in terms of their geographical location as well as the magnitude of their scale, we opted for a simple way of grouping the different regions. Fourteen databases compile information at a worldwide level (37.8%), twenty-one databases assess smaller regions, here designated as non-worldwide (56.7%), and which may vary from countries such as Canada or Japan, to the Mediterranean Sea or Europe. The two remaining databases do not clearly describe the region from which the traits were compiled.

Regarding the number of taxa described in each database, sixteen databases had between 50 and 250 taxa and only six databases described 251–500 taxa, which together accounted for 59.4% of all studies. Five databases had between 501 and 1000 taxa, four between 1001 and 2500 and seven with more than 2500. We divided the Freshwater Ecology traits database^22^ in two subgroups to calculate these taxa number intervals because their data for fish and invertebrates is isolated from each other, allowing for a clearer insight on trait data availability regarding the taxa groups.

**Figure 2:**
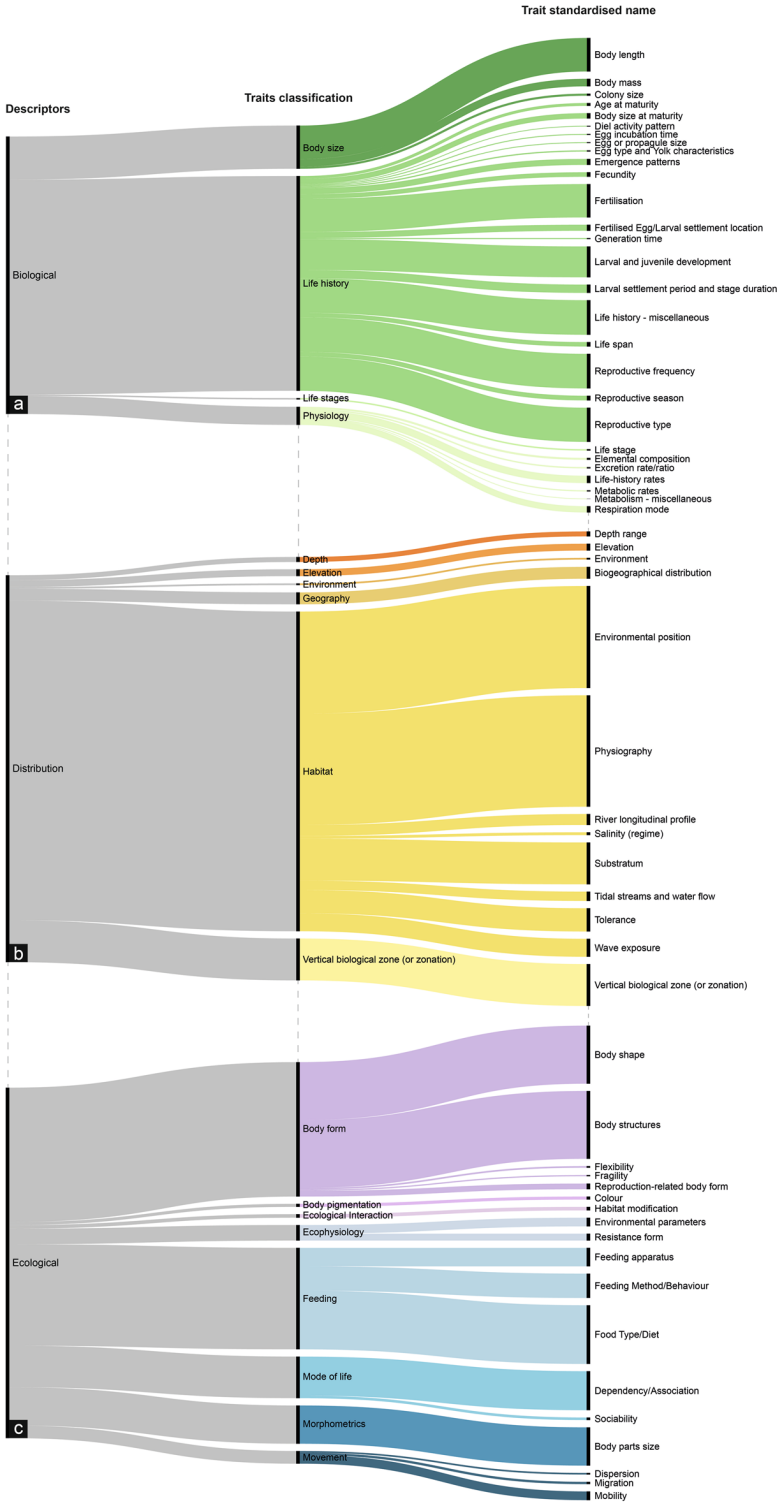
Distribution of the maximum number of taxa registered and described for each Trait standardised name over the three hierarchical levels from our unified nomenclature proposal: Trait descriptors, Traits classification and Trait standardised name. For clarity, data were divided into three alluvial diagrams corresponding to the three Trait descriptors: Biological (**a**), Distribution (**b**) and Ecological (**c**). Colours are the same as in Fig. 1 and correspond to the different Traits classifications. The size of the bars is proportional to the number of studies for which databases containing information on each hierarchical level of traits are available.

A total of 1127 traits were collected and assigned to three main groups, 475 Biological (42.2%), 162 Distribution (14.4%) and 490 Ecological (43.4%) (Fig. 1). The three largest trait classifications were Life history (n = 253, 22.45%), Body form (n = 115, 10.20%) and Physiology (n = 109, 9.67%). Overall, the three most common traits are Body parts size (n = 99, 8.78%), Body length (n = 82, 7.28%) and Body structures (n = 80, 7.10%). Within the Biological descriptors, Body length was the most common trait (n = 82, 7.28%), followed by Fecundity (n = 36, 3.19%) and Elemental composition (n = 32, 2.84%). The most abundant Distribution traits were Substratum (n = 22, 1.95%), Depth range (n = 21, 1.86%) and Biogeographical distribution (n = 20, 1.77%). Finally, Body parts size (n = 99, 8.78%) was the largest classification under Ecological traits, followed by Body structures (n = 80, 7.10%) and Environmental parameters (n = 68, 6.03%) (Fig. 2).

Specifically for the fish group, 507 fish traits were gathered from datasets exclusively focused on fish taxa, from which roughly half were categorised as Ecological (n = 281, 55.4%), followed by Biological (n = 171, 33.73%) and Distribution (n = 56, 11.05%). The largest trait classification belonged to the Biological group, where 107 traits were classified as Life history (21.10%), followed by 95 Morphometrics traits (18.74%) and 78 Body form (15.38%). Body parts size (n = 95), Body structures (n = 65) and Body length (n = 45) were the most common traits, and Physiography (n taxa = 31,369), Body shape (n taxa = 17,556), and Body structures (n taxa = 14,173) were the traits with the highest number of taxa.

We found 390 traits accessible in invertebrates’ datasets, the majority of which equally distributed between Ecological (n = 157, 40.26%) and Biological (n = 154, 39.49%), with the remaining traits falling within the Distribution group. Similarly, to the fish traits described above, Life history was the classification with the largest number of traits (n = 98, 25.13%), followed by Ecophysiology (n = 46, 11.79%) and Habitat (n = 45, 11.54%). Despite being the main group with the lowest trait number, Distribution presented the highest number of taxa described for invertebrates (Habitat—3606; Geography—3339). The third and fourth traits with the highest number of taxa were Feeding (2891) and Body form (2473), both belonging to the Ecological descriptors.

Zooplankton datasets described 116 traits which present a different distribution when compared with the other taxa groups. The vast majority was grouped as Biological (n = 103, 88.79%), where Physiology (n = 61, 52.59%), Body size (n = 23, 19.83%) and Life history (n = 19, 16.38%) were the largest trait classifications. The rest of the traits were distributed along the Ecological (n = 8, 6.9%) and Distribution descriptors (n = 5, 4.31%). Body size, more specifically Body length (1850), had the highest number of taxa described, followed by two Ecological traits, Environmental position and Biogeographical distribution, both described for 701 different taxa.

Lastly, 114 traits were described in datasets which combined more than one taxonomical group (more specifically, datasets of Fish, Invertebrates and Fish, Invertebrates, Zooplankton). With a trait distribution along the three groups similar to the one seen in invertebrates, where the majority of traits were grouped under Biological (n = 47, 41.23%) and Ecological (n = 44, 38.60%). The three largest trait classifications were Life history (n = 29, 25.44%), Habitat (n = 14, 12.28%) and Ecophysiology (n = 12, 10.53%), with Environmental parameters (n = 12, 10.53%) being the most common trait, followed by Food Type/Diet (n = 8, 7.02%) and Elemental composition (n = 6, 5.26%). Overall, there were no discrepancies in standardised trait numbers, as they displayed similar frequencies within each trait classification. However, the traits with data for the largest taxa number were Environmental position (28741), Body structures (20518) and Food Type/Diet (17743).

### Ecological relevance of the traits

In addition to the main set of traits available based on their abundance and number of taxa described, we briefly assessed their availability according to their ecological meaning. We adapted^13^ unified typology of aquatic functional traits, in which they established a link between trait type (Morphological, Life history, Physiological and Behavioral) and an ecological function (Resource acquisition, Growth, Reproduction and Survival). We can also analyse it considering the trait reflects the fitness of the species under given environmental conditions (i.e., response trait) or influences an ecological process (i.e., effect trait)^9^. In order to transpose trait data availability to a potential ecological meaning, we determined the traits with the most taxa descriptions for each group and established a correspondence adapting^13^ diagram. Nevertheless, it is worth noting that these traits do not necessarily reflect an ecosystem function when analysed alone^9^. Frequently, a combination of different traits or even their diversity is key for assessing an ecosystem function, which in turn is highly context dependent^9,20,46^.

**Figure 3:**
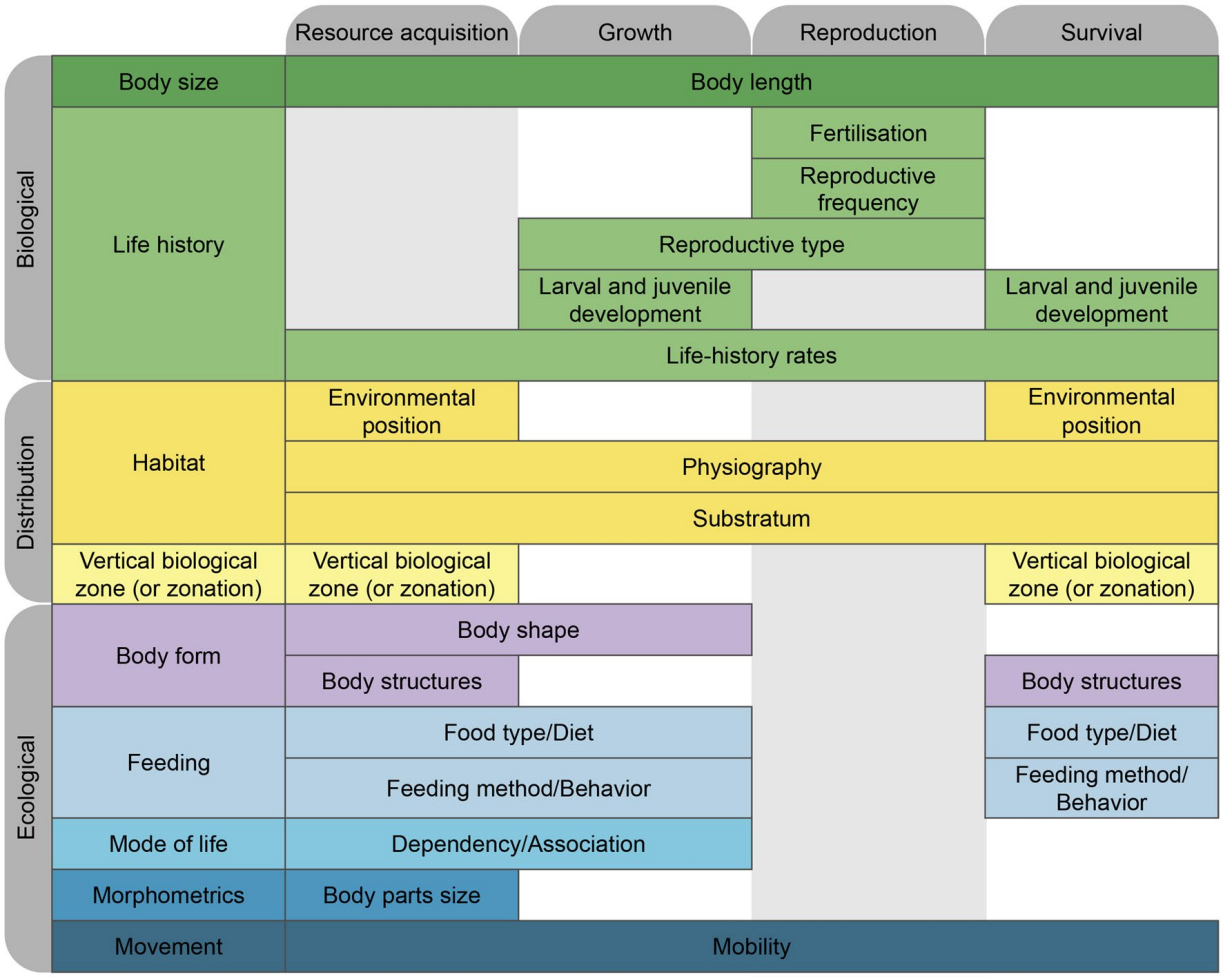
Overview of available traits to measure ecological functions by adapting the unified typology on aquatic functional traits proposed by^13^ to the identified standardised trait names with most data described available according to this study.

First, the most abundant Biological traits were Body length, Fertilisation, Reproductive frequency and type, larval and juvenile development and life history rates (Fig. 2a), which successfully correspond to the four ecological functions mentioned above (Fig. 3).

Body size, measured as body length, is one of the most common and measurable traits across biological groups, often considered a key trait influencing the dynamics and structure of aquatic communities. It influences energy requirements, food-web and biotic interactions, metabolic rates and dispersal^29,40^, thus relating to all functions (Fig 3). The life-history rates from the physiology group, collectively combining rates from various processes/functions, including growth itself, also relate to all functions (Fig. 3), by influencing species' performance or fitness against an environmental background. Fertilisation and reproductive frequency relate to reproduction but may also influence dispersal abilities, whereas larval and juvenile development provides information on both growth and survival.

Secondly, the most common Distribution traits were Environmental position, Physiography, Substratum and Vertical Biological Zone (Fig. 2b), all matching ecological functions (Fig. 3). These traits are usually considered as proxies of the actual trait that allows a species to distribute in an environment or habitat, as mentioned above. Yet, those proxies are often used since it is easier to get information. Physiography and the Substrate relation may be used to infer conclusions for all four ecological functions as broadly reflecting the environment where the species occurs and develop, including habitat preferences (Table 2). Information on the Environmental position and Vertical Biological Zone is often mixed in literature, as they reflect the species position either relative to the water column or substratum (i.e., environmental position) or wider zonation patterns (details in *Standardized_traits* dataset), which are potentially related to resource acquisition or survival (Fig. 3).

Finally, the five most abundant Ecological traits are as follows: Body parts size, Body structures, Environmental parameters, Food Type/Diet and Mobility (Fig. 2c). These correspond with multiple sections of the diagram, exhibiting potential links to all four ecological functions, with a higher number of overlaps for resource acquisition and lower for reproduction (Fig. 3). There can be also different interactions between these traits. For instance, mobility can be expressed through morphometric measurements or body shapes, while mobility itself is related to all functions and also influences dispersal abilities.

### How to select traits for a given study & good practices

**Figure 4:**
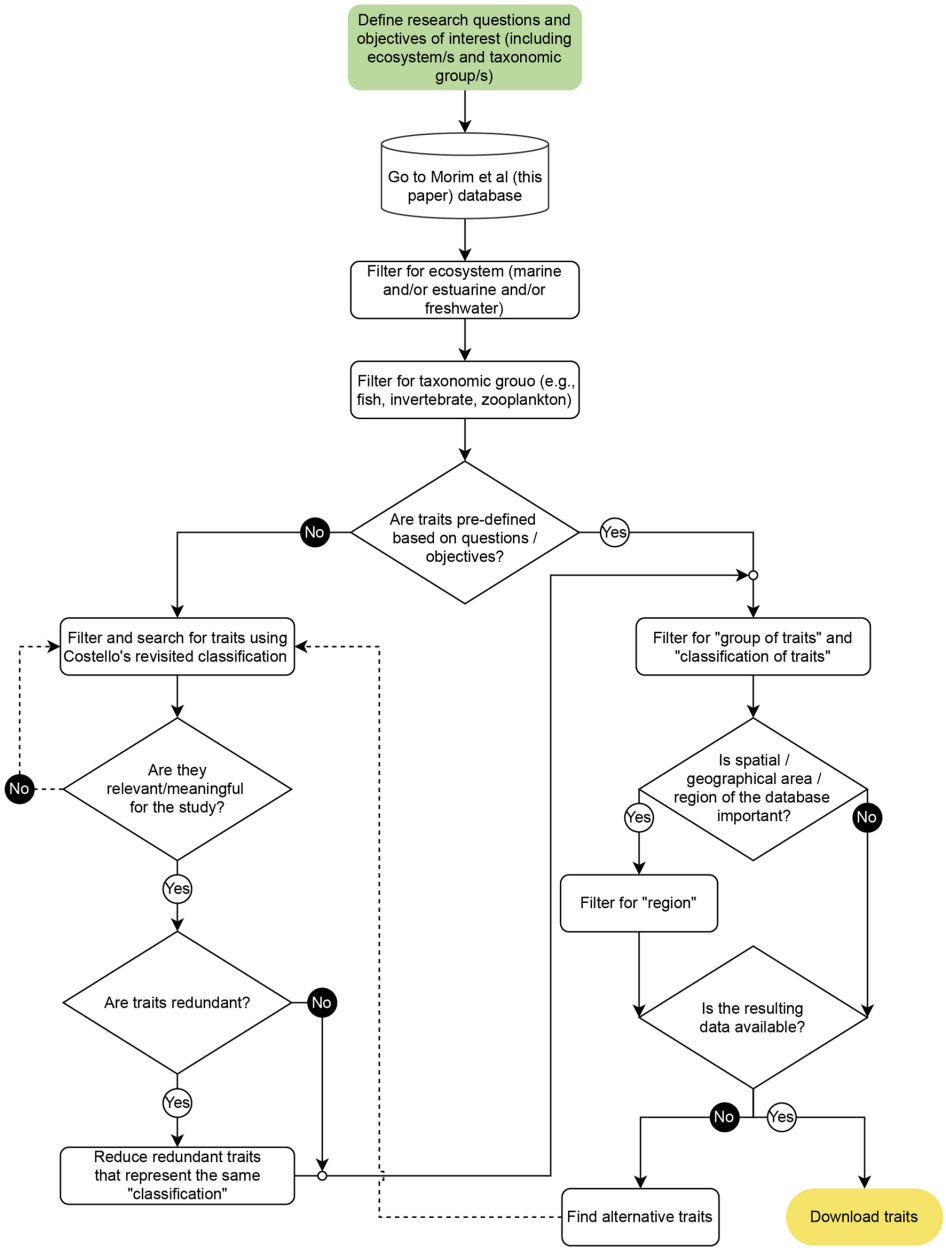
Roadmap describing the main steps and questions when choosing a set of traits for a functional diversity study. At least four main points should be addressed: deciding the target ecosystem, target taxonomic groups, study’s scale and testing for trait redundancy.

The trait selection process necessary to navigate when starting a study is equally complex as it analyses^25^ and of utmost importance, as it will directly influence the study’s results. Here, we provide a roadmap (Fig. 4) that aims to facilitate this process by guiding the user through a series of steps and answering a few crucial questions. The roadmap selection was built considering the data availability under this review, where available information can be accessed using the *Trait_Sources&Measurements* dataset filter selection.

Briefly, the first step should be defining the research questions, objectives, and testing hypotheses. An initial framework for the steps involved in setting the hypotheses based on effect or response traits is available in^9,47^. Then, one should consider whether it is a large- or local-scale study by selecting the ecosystem type and whether the focus is on a particular taxa group of interest, as both strongly influence the data availability. For high diversity studies (e.g., large scale, diversity hotspots), comprising multi-trophic taxa groups, the traits selection should focus on those with the most information available (Fig. 2) that can still inform the researcher's questions (Fig. 3). Still, the trait range values may change for each of these trophic groups or even for different taxa within each trophic group. Specifically, multi-trophic studies may consider these groups as compartments to calculate their functional properties, or to consider a multitrophic response-effect framework to include their interactions (more details in^9^).

For other studies, researchers can select other particular traits fitter for specific questions or even consider measuring them directly (realised traits^13^). Once traits suitable to answer the research questions are chosen, one must check for trait redundancy, as different traits serve as a proxy for the same functions and analyses will benefit from a lower number of traits with low levels of redundancy (e.g., increasing the number of traits in the analysis can lead to an artificial increase of differences in functional space or mask functional trait patterns and response to changes). If present, redundant traits should be removed, simplifying the set of chosen traits. The one to choose should rely on traits with precise and interpretable functional relationships for the research question and data availability for the species' dataset and region, with priority given to standardised measurements and to the easiest to interpret. Selection could also rely on adding traits only when they provide new information or applying model selection approaches or dimensionality reduction techniques. However, if relevant to the aim of the study, redundancy relationships can be taken into account when interpreting the results, but should be avoided to run the analyses (e.g. estimation of functional diversity indices, to modelling trait response).

In case the scale of the study/target region is relevant to the research questions, one should filter for the region when checking for data availability of the already selected traits. The final step of this process would be downloading the available trait data. However, these questions and line of flow should be repeated if data for selected traits are unfortunately unavailable in order to find a new suitable trait. It is also important to note that most of the information available in the literature reflects an average value for the species in its environment unless it is clearly assumed that the trait measurements also include intraspecific variability. Indeed, most studies of functional diversity assume that interspecific variability is greater than intraspecific variability. Nowadays, however, the importance of this intraspecific or within-species variability is increasingly recognised, including methods to quantify it^9,48^. If one is interested in quantifying this aspect as well, we primarily advise to either specifically look for all information available for the species (e.g. phenotypic plasticity or trophic plasticity) or actually measure a number of individuals for each species (e.g. size), which in some cases may be the only viable option.

Finally, it is difficult to compare functional diversity (whether indices or trait identity responses) between studies when different traits and different number of traits are selected, not to mention monitoring schemes. This is exacerbated when the same traits are labelled differently. Standardising the nomenclature, as proposed here, and this roadmap for selecting non-redundant traits would thus be a first step towards a common framework to guide the selection of the most relevant traits for the specific aim of the study. This is particularly relevant for meta-analyses, as having the synonyms and definitions would increase the number of potential studies that could be included in meta-analyses. To support this functional diversity studies comparability, we also advise new trait studies to always include the data source used to classify each trait (rather than generic information) and the traits data as an appendix (for aggregated traits, the information of each individual trait should be presented) so that other authors can compare the trait response.

### Future directions

Trait-based studies are rising in popularity, thanks to the overall perception of their capability to inform on the relationship between individuals and the environment. Advances in computational techniques and better knowledge of species ecology^9^ have led to a large number of researchers currently utilising traits in their studies. The number of published trait data is increasing and new databases are frequently released, some of which were not available at the beginning of this work and, thus, have been not included (e.g.,^49,50^). We emphasize the need to build on this work, which clarifies the traits' possible measurements, definitions and ecological relevance, for comparable trait studies. However, data collection will certainly benefit from being revisited in a few years. Since 2020, the databases available may have already been updated, resulting in changes in the array of traits accessible and on taxa number counts (e.g.^23,33^). Although our proposed nomenclature considers the current Wiki version of the^33^, their website now includes a list of links with existing initiatives (e.g.^21,23,51^) whose information is now also linked under their dataset to avoid online data duplication. We maintain these databases in our data compilation as they provide a valuable and significant source of information that was still being implemented within the^33^ when this work started and because they will be useful for past published studies using these datasets. Also, other authors may still be using them in the future, as they are widely known in the scientific community for specific biological targets (e.g., fish^23^). Nevertheless, we acknowledge how essential the efforts of these data compilations are and must emphasise that retrieving data from singular datasets may soon become obsolete.

Another important aspect is the availability of multiple versions of the same dataset and how they tend to be utilised by the community. To avoid the pseudoreplication of data, we only included complete datasets in their most up-to-date version, simultaneously acknowledging this would result in failing to include a small amount of published data. However, not every study will prefer to use the original/complete dataset. Researchers will often analyse subsets of larger databases, sometimes manually adding trait data from the literature to fill in information gaps on their target taxa, thus making available smaller subsets that include additional data originally unavailable. ^52^ are one of these cases, where the FishBase subset they published is often preferred and analysed instead of the original data source because the data is now reorganised, potentially more complete and easier to access/use. The effort necessary to compare and isolate the differences between an original dataset and its subsets is extremely elevated, and projects seldom can do this type of maintenance and update regularly. This results in increased inaccessibility of small datasets freshly compiled for small-scaled compilations such as this work, and further evidences the urgent need for creating collaborative efforts in the compilation and processing of data.

Finally, this study has clearly expressed the need for unifying traits’ names and definitions, as many terms retrieved directly from the literature were ambiguous in their meaning and use. Being able to search, find and correctly employ traits thus relies on having a scientific community in agreement regarding trait nomenclature. Moreover, in a field of study where the array of traits assessed directly affects the outcome of our research, a clear understanding of the information traits carry is essential to obtain effective answers for our problems and hypotheses.

### Supplementary Information

Supplementary Information 1.

Supplementary Information 2.

Supplementary Information 3.

## Data Availability

All data generated or analysed during this study are included in this published article [and its supplementary information files]. The two datasets in the supplementary information are also available at the following link: https://figshare.com/s/656d00772224c01dc3f1.
