# Author Correction: A roadmap to define and select aquatic biological traits at different scales of analysis

**DOI:** 10.1038/s41598-024-51894-y

**Published:** 2024-01-17

**Authors:** Teófilo Morim, Sofia Henriques, Rita Vasconcelos, Marina Dolbeth

**Affiliations:** 1 CIIMAR - Interdisciplinary Centre of Marine and Environmental Research, Novo Edifício do Terminal de Cruzeiros do Porto de Leixões, Avenida General Norton de Matos s/n, 4450-208 Matosinhos, Portugal; 2 IPMA – Instituto Português do Mar e da Atmosfera, Av. Dr. Alfredo Magalhães Ramalho 6, 1495-165 Algés, Portugal; 3 MARE - Marine and Environmental Sciences Centre & ARNET – Aquatic Research Infrastructure Network Associated Laboratory, Faculdade de Ciências da Universidade de Lisboa, Campo Grande, 1749-016 Lisbon, Portugal

Correction to:* Scientific Reports* 10.1038/s41598-023-50146-9, published online 22 December 2023

The original version of this Article contained an error in the Acknowledgements section.

“This work was funded by Ocean3R (NORTE-01-0145-FEDER-000064), supported by the North Portugal Regional Operational Program (NORTE2020), under the PORTUGAL 2020 Partnership Agreement and through the European Regional Development Fund (ERDF) and PTDC/BIA-ECO/29261/2017 by Fundação para a Ciência e a Tecnologia (FCT). TM was supported by FCT through PTDC/BIA-ECO/29261/2017, SH by Portuguese National funds of FCT through a research contract DL57/2016/CP1479/CT0021 and UID/MAR/04292/2019, RV by the ‘Programa Nacional de Amostragem Biológica’ (EU Data Collection Framework) and MD by FCT CEEC contract CEECINST/00027/2021/CP2789/CT0001), by the European Union’s Horizon 2020 research and innovation programme under grant agreement No 869300 "FutureMARES" and by the Strategic Funding UIDB/04423/2020 and UIDP/04423/2020 through national funds provided by FCT and ERDF.”

now reads:

“This work was funded by Ocean3R (NORTE-01-0145-FEDER-000064), supported by the North Portugal Regional Operational Program (NORTE2020), under the PORTUGAL 2020 Partnership Agreement and through the European Regional Development Fund (ERDF) and PTDC/BIA-ECO/29261/2017 by Fundação para a Ciência e a Tecnologia (FCT). TM was supported by FCT through PTDC/BIA-ECO/29261/2017, SH by Portuguese National funds of FCT through a research contract DL57/2016/CP1479/CT0021 and by the Strategic Funding UIDB/04292/2020 and UIDP/04292/2020, RV by the ‘Programa Nacional de Amostragem Biológica’ (EU Data Collection Framework) and MD by FCT CEEC contract (CEECINST/00027/2021/CP2789/CT0001), by the European Union’s Horizon 2020 research and innovation programme under grant agreement No 869300 "FutureMARES" and by the Strategic Funding UIDB/04423/2020 and UIDP/04423/2020 through national funds provided by FCT and ERDF.”

The original Article has been corrected.

