# Supplementary material for: A roadmap to define and select aquatic biological traits at different scales of analysis
Source: Sci Rep. 2023 Dec 22;13:22947. doi: 10.1038/s41598-023-50146-9 (PMC10746726; doi:10.1038/s41598-023-50146-9)
Supplement: Supplementary file 3 — Supplementary Information 3. [file 41598_2023_50146_MOESM3_ESM.docx]

# Supplementary material

*Trait_Sources&Measurements* dataset: dataset with standardized trait names organized according to hierarchical trait grouping and classification based on traits databases review, including identification of main environment, taxa group, trait original name and definition of author, number of taxa described, type of measurement and units or modalities, name of author, name of database (if available), accessibility, and literature source.

*Standardized_traits* dataset: dataset containing the standardized names of the traits, ordered by hierarchical grouping and classification, with their respective definitions, synonyms found in the literature, measurement options found in the database and those mentioned in the Marine Species Traits Wiki, the literature source of the definition, the number of databases available for each trait, and the minimum and maximum number of taxa described. (Note: This dataset is the expanded version of Table 2 from the main text, which builds on the Trait_Sources&Measurements dataset).
